# Hemolytic uremic syndrome in severe acute pancreatitis

**DOI:** 10.1093/omcr/omaf048

**Published:** 2025-05-28

**Authors:** Jayaraj Hasvi, Inder Preet Singh Bhatia, Saurabh Dawra, Amulyajit Singh, Siddharth Tripathi

**Affiliations:** Department of Internal Medicine, Military Hospital, Bareilly 243001, Bareilly district, India; Department of Internal Medicine, 167 Military Hospital, Pathankot 145001, Pathankot district, India; Department of Gastroenterology, Command Hospital Udhampur 182101, Udhampur district, India; Department of Radiodiagnosis, PGI, Jalandhar 144003, Jalandhar district, India; Department of Internal Medicine, 167 Military Hospital, Pathankot 145001, Pathankot district, India

**Keywords:** Hemolytic uremic syndrome, severe acute pancreatitis, plasmapheresis, intravenous immunoglobulin, rituximab

## Abstract

Introduction: Hemolytic Uremic Syndrome is characterized by Coombs negative microangiopathic hemolytic anemia, severe thrombocytopenia, and acute kidney injury.

Case Report: Here, we report a 36-year-old male, a case of alcohol related acute pancreatitis. On day 5, he developed fever and tachycardia. Evaluation revealed thrombocytopenia, hemolytic anemia, and acute kidney injury with low C3, normal C4 & ADAMTS13 activity and positive anti-complement factor H antibody. He was diagnosed as a case of Hemolytic Uremic Syndrome and managed with plasmapheresis, hemodialysis, and Intravenous immunoglobulin. He was administered Rituximab on day 15, considering the refractory nature of the disease. Despite the aggressive management, he succumbed to his illness.

Conclusion: This case highlights atypical Hemolytic Uremic Syndrome as a rare complication of Acute Pancreatitis. Our patient, despite being diagnosed well in time and managed aggressively, had an unfavourable outcome. This condition should be diagnosed well in time, as it is associated with high mortality.

## Introduction

Acute Pancreatitis (AP) is an acute inflammatory process of the pancreas which can initiate a systemic inflammatory response syndrome (SIRS) resulting in acute respiratory distress syndrome (ARDS) and multi organ dysfunction. Common etiologies include gall stones, alcohol, hypertriglyceridemia, Endoscopic retrograde cholangiopancreatography, drugs, trauma, post-operative. Uncommon causes include hypercalcemia, infections, cystic fibrosis, autoimmune and hereditary causes. It is associated with various hematological complications like fall in serial values of hemoglobin, coagulation abnormalities, disseminated intravascular coagulation (DIC), acute hemolytic anemia, thrombocytopenia and thrombotic thrombocytopenic purpura or hemolytic uremic syndrome [1]. Hemolytic Uremic Syndrome (HUS) secondary to Acute Pancreatitis is a rare presentation [2] and total of 38 cases have been reported in the past [3–6]. HUS is characterized by a Coombs negative microangiopathic hemolytic anemia (MAHA), severe thrombocytopenia and acute kidney injury (AKI), and is associated with high mortality [7]. Hence, prompt diagnosis and timely initiation of appropriate management would result in favourable outcome.

## Case report

36-year-old male, known case of pulmonary tuberculosis (2018, completed ATT) presented with a history of severe epigastric and left hypochondriac pain of 01-day duration, which was radiating to the back, relieved on bending forward. The patient gave a history of consumption of alcohol 2 days before the onset of symptoms. At the time of presentation, the patient gave no history of fever, palpitations, breathlessness, decreased urine output, any bleeding manifestations, any chest pain, breathlessness, cough, or hemoptysis.

On examination, his vitals (at admission) included a pulse of 84/minute, a blood pressure of 108/90 mm of Hg, a temperature of 98.7°F, respiratory rate of 18/minute and oxygen saturation of 97% at room air. There was no evidence of pallor, icterus, clubbing, lymphadenopathy or edema. Abdominal examination revealed tenderness over the left hypochondrium, with decreased bowel sounds. The rest of the general and systemic examination was normal, without any evidence of hepatosplenomegaly.

On further evaluation, he was found to have increased amylase (1575 IU/l) and lipase (12 500 IU/l) and Ultrasound abdomen was suggestive of Acute Pancreatitis. The rest of the hematological and biochemical parameters were normal. He was initially managed with parenteral fluids, analgesics, proton pump inhibitors and supportive care.

**Figure 1:**
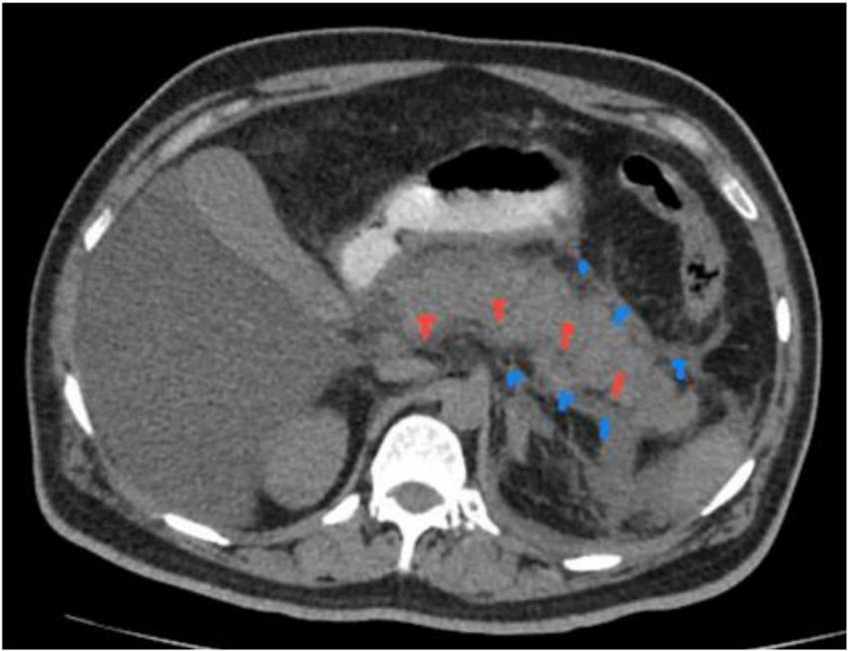
NCCT with oral contrast. It reveals bulky pancreas, peripancreatic fluid and fat stranding.

**Figure 2:**
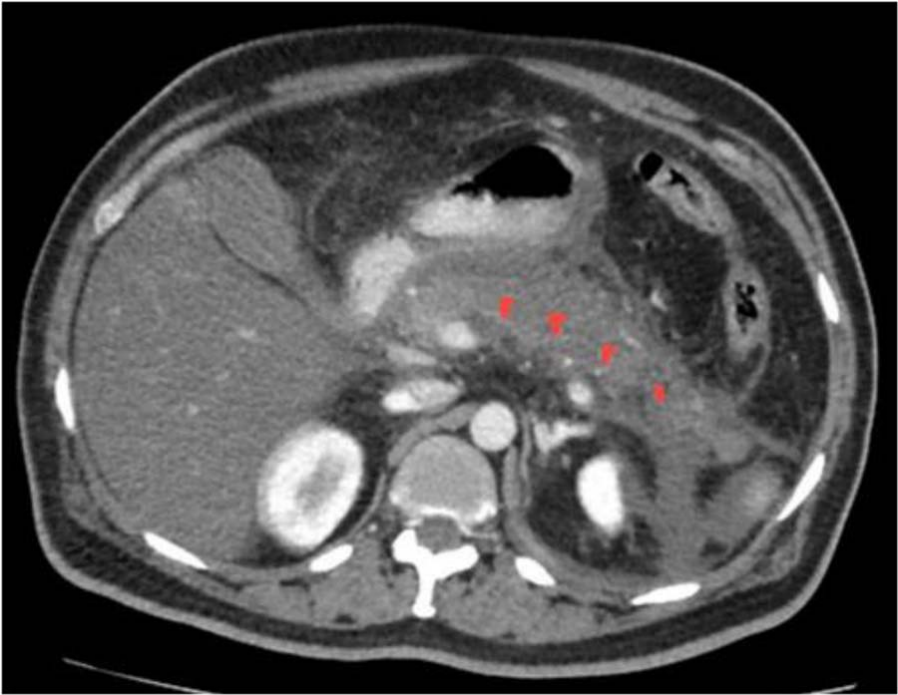
CECT with pancreatic phase. It reveals non enhancement of body and tail of pancreas, indicating necrotizing pancreatitis.

**Figure 3:**
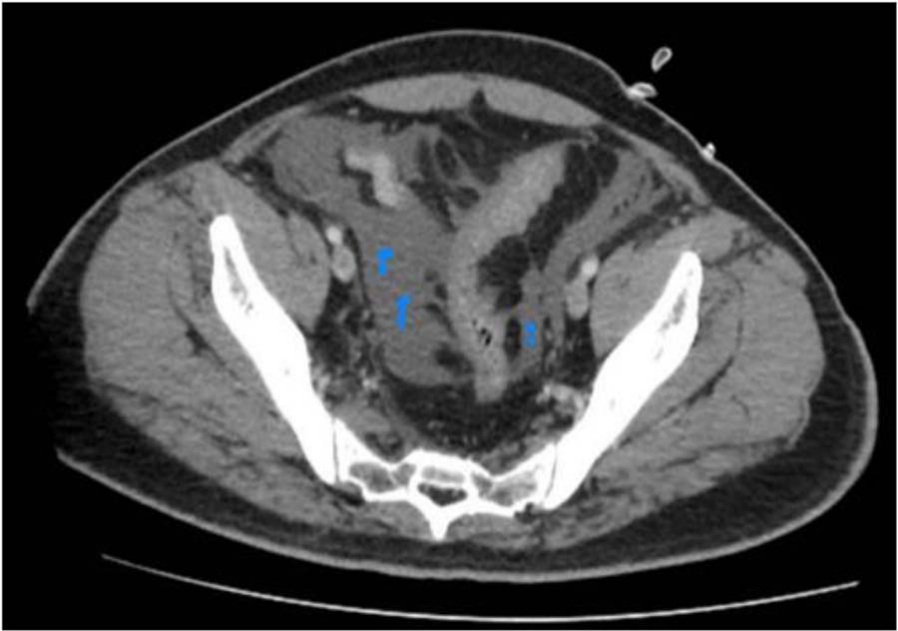
Further caudal scans reveal ascites.

**Figure 4:**
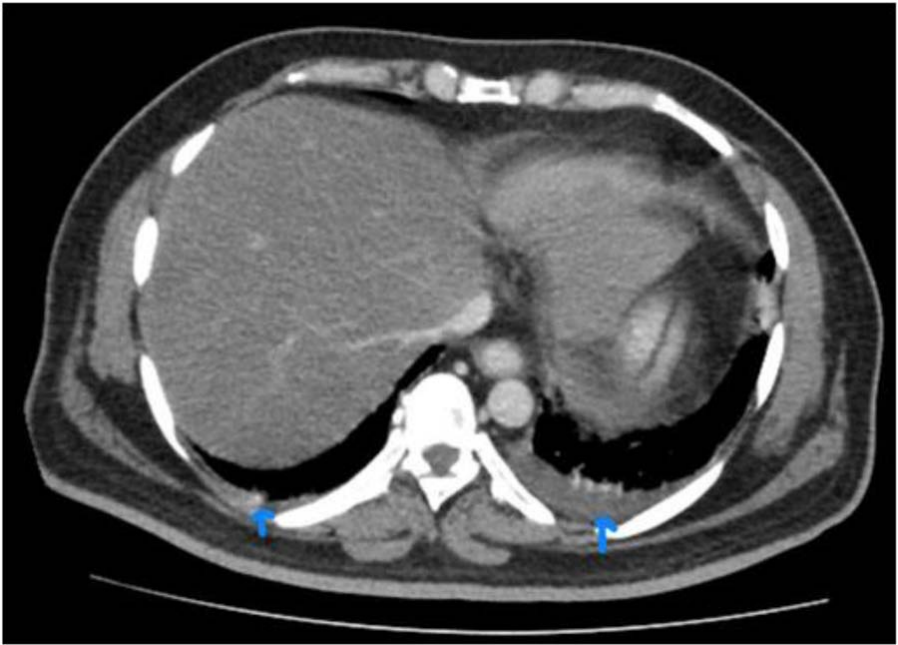
CECT abdomen with visualized chest cuts in the same patient indicating bilateral pleural effusion.

He underwent a CT abdomen on day 3 of admission, which revealed Acute Necrotizing Pancreatitis, with a modified CTSI score of 10/10 (Figs 1–4).

On day 5 of admission, the patient developed fever. Evaluation revealed tachycardia (heart rate − 124/minute) with neutrophilic leucocytosis (total leucocyte count of 28 300; polymorphs- 94%, lymphocytes- 2%). He was also found to have a drop in haemoglobin (13.3 to 8.8 gm/dL) with thrombocytopenia (Platelets—1,75 000 to 29 000/mm^3^). Peripheral blood smear (PBS) showed toxic changes along with the presence of schistocytes (3.5%). Direct coombs test (DCT) and indirect coombs test (ICT) were negative. His serum procalcitonin was elevated (58.01 ng/ml, normal < 0.1 ng/ml). Renal parameters were found to be deranged (Urea/creatinine- 43/1.0 to 74/3.5 mg/dl) with abnormal liver function tests (bilirubin-1.3/0.9 to 4.8/1.2 mg/dl) and raised serum lactate dehydrogenase (LDH) (181 to 1251 IU/l). He was started on plasmapheresis (considering the possibility of hemolytic uremic syndrome), parenteral empirical antibiotics and was transfused with packed red blood cells and continued on parenteral fluids & other conservative measures.

On further evaluation, it was found that hemolytic anemia was pertaining to be complement mediated [decreased C3, normal C4, anti-compliment factor H antibody-positive; 298.35 AU/ml (0–150)]. ADAMTS13 activity was normal. He was managed as a case of Severe Acute Pancreatitis (Alcohol related), Atypical HUS with plasmapheresis, Intravenous Immunoglobulin (IVIg) (2 gm/kg body weight over 5 days) and alternate day hemodialysis. The patient had no response to the plasmapheresis & IVIg, continued to have schistocytes on PBS, thrombocytopenia and elevated LDH. He was administered Rituximab 675 mg (500 mg/m^2^) on day 15 of admission, considering the refractory nature of the disease. Despite intensive interventions, his condition continued to deteriorate, and he ultimately succumbed to his illness on the 21^st^ day of admission.

**Figure 5:**
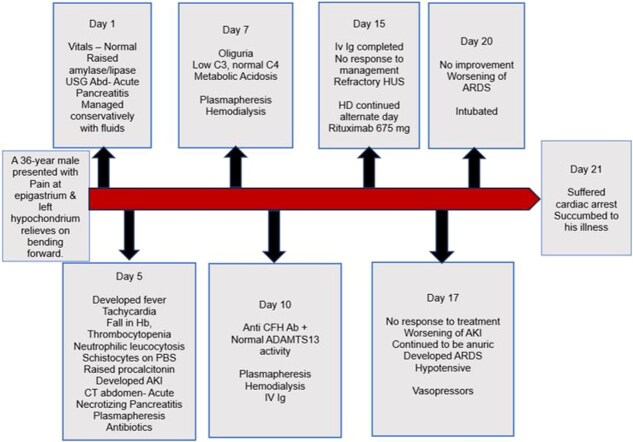
Timeline of events.

The timeline of the events and lab parameters of this case report have been summarized in Fig. 5 and Table 1 respectively.

**Table 1:** Lab parameters.

Lab	On	Day 5	Day 07	Day 10	Day 15	Day 20
Parameters	admission					
Hb g/dl	13.3	8.8	8.2	7.7	8.3	7.8
TLC/uL	9300	28 300	22 300	19, 400	17 500	22 500
DLC	N-78%,	N- 94%	N- 89%	N- 90%	N- 86%	N- 92%
	L- 18%	L- 02%	L- 07%	L—07%	L – 06%	L – 04%
Platelets 10^3^/ul	1,75 000	29 000	36 000	32 000	48 000	38 000
PT	14.2	14.8	16.4		17.5	
INR	1.0	1.1	1.2		1.3	
aPTT	34	33	35		36	
Urea/Creatinine mg/dl	43/1.0	74/3.5	89/5.5	112/7,2	138/7.9	170/9.2
Na^+^/K^+^ mEq/L	137/3.9	142/4.2	137/5.3	140/5.9	138/5.4	135/4.9
Ca^2+^/Po4^3−^ mg/dl	9.2/2.9	8.7/3.5	9.0/3.7	8.6/5.5	8.9/4.1	9.1/4.7
Bilirubin (D/I)	1.3/0.9	4.8/1.2		3.8/0.9		
mg/dl						
SGOT/SGPT	37/49	327/816		180/550		
IU/L						
ALP/GGT	110/278					
Amylase	1575					
Lipase	12 500					
Procalcitonin		58.01		28.7		
Serum LDH	181	1251		1130	1470	
Serum fibrinogen				250		
200–500 mg/dl						
D-Dimer ng/ml				780		
Urine C/S			No growth			
Blood C/s				No growth		
ABG						
pH	7.37	7.29	7.22	7.18	7.15	6.93
pCo2 mm Hg,	38	36	30	24	20	18
HCO3^−^ mmol/L	21	20	17	12	09	05
HbsAg		Negative				
Anti HCV		Negative				
HIV		Negative				
C3 mg/dl (82–160)			47			
C4 mg/dl (12–36.0)			28			
Anti CFH Ab AU/mL				298.35		
by ELISA (0–150)						
ADAMTS13 Activity				110%		
(60–130)						

## Discussion

AP is an acute inflammatory process of the pancreas that can trigger a systemic inflammatory response. It is associated with various hematological complications like serial fall in hemoglobin, coagulation abnormalities, disseminated intravascular coagulation, acute hemolytic anemia, thrombocytopenia, and thrombotic thrombocytopenic purpura or hemolytic uremic syndrome [1].

HUS is a rare disease characterized by a Coombs negative microangiopathic hemolytic anemia (MAHA), severe thrombocytopenia and AKI [8]. The main pathology is thrombotic microangiopathy associated with endothelial cell injury and platelet activation, which mainly affects the kidney, as well as other organs [9]. HUS is usually categorized as typical, caused by Shiga toxin-producing *Escherichia coli* (STEC) infection and as atypical HUS (aHUS), usually caused by complement dysregulation due to mutations or autoantibodies, disorders of the degradation of von Willebrand factor (VWF), or secondary to a coexisting condition like cobalamin metabolism disorders, pregnancy/HELLP Syndrome, certain drugs (Chemotherapeutic drugs, immunosuppressants, antiplatelets etc) and other disorders (e.g. systemic diseases appearing as HUS, such as systemic lupus erythematosus and rejection after transplantation) [8]. These share a common pathogenetic mechanism of complement hyperactivation causing a prothrombotic and proinflammatory state on the endothelial cell surface, leading to endothelial damage and microvascular thrombosis [10].

Atypical HUS itself, is extremely rare, seen in adults with an incidence of 1 to 7 per 1 000 000 in the U.S. and Europe [7]. aHUS triggered by pancreatitis is definitely a very rare complication. Atypical HUS secondary to AP has previously been published in only a few case reports. It is generally accepted that pancreatitis can occur in TTP-HUS. But the occurrence of AP preceding the development of HUS with a short interval (1–13 days; median, 3 days) between the onset of pancreatitis and the development of HUS, suggests that the inflammatory response (secondary to pancreatitis) leading to endothelial damage is causally associated with the pathogenesis of TTP-HUS. The systemic inflammatory response mediated by IL-6, IL-8, TNF-α, and other cytokines, contributes to the development of HUS in Acute Pancreatitis [11].

Outcome is mostly good in childhood onset, Shiga toxin-associated HUS. But the renal complications are more common in adult, atypical and familial forms of HUS with a high mortality rate as well. End-stage renal disease (ESRD) or death occurs in about 33% to 40% of patients during the first clinical manifestation of atypical HUS. Within 1 year after a diagnosis of aHUS, up to 65% of patients treated with plasma exchange or infusion develop permanent renal damage and progression to ESRD, or die [12].

The treatment of HUS following AP is primarily supportive. Exchange plasmapheresis has been reported to be effective, with a response rate of 79% [13]. Rituximab (anti-CD20 antibody) is beneficial in refractory or relapsing HUS [14].

Long-term treatment with the anti-C5 monoclonal antibody, Eculizumab has been proven to be effective in aHUS. Furthermore, the current evidence suggests that Eculizumab, is effective in both aHUS and STEC-HUS and may have some benefit in TTP as well [10]. Eculizumab, a terminal complement inhibitor, is a humanized monoclonal antibody that acts by binding to the human C5 complement protein, with high affinity and blocks the generation of proinflammatory C5a and C5b-9. Thereby, inhibiting the complement-mediated thrombotic microangiopathy, decreases the need for thrombotic microangiopathy–related interventions. It also significantly improves the platelet count and renal functions. The earlier the intervention, the greater will be the clinical benefits.

Our case presented with features suggestive of AP. He underwent CT abdomen, findings of which were suggestive of Acute Necrotizing Pancreatitis, with a modified CTSI score of 10/10. On day 5, the patient developed features of SIRS. He was found to have neutrophilic leucocytosis with significant schistocytes on PBS. Furthermore, he was found to have raised procalcitonin, AKI, indirect hyperbilirubinemia and raised serum LDH. He was started with plasmapheresis on day 5 based on high suspicion of HUS. Further evaluation revealed low C3 levels, normal C4 levels with positivity for anti-Complement Factor H antibody. He was diagnosed with Atypical HUS. In addition to Plasmapheresis, IVIg (2 gm/kg body weight over 5 days) and alternate day hemodialysis were initiated. He received total of 8 sessions of plasmapheresis and considering the refractory nature of the disease, he was administered Rituximab on day 15 of admission. DIC was also considered as differential diagnosis, but normal fibrinogen, near normal values of D-Dimer & INR ruled out the possibility of DIC. Because of non-availability of eculizumab in our centre, this patient was managed with other modalities, initially with plasmapheresis (as it remains the first line of management at the centre where Eculizumab is not available), followed by plasmapheresis with IVIg and then Rituximab in view of refractory disease. There was no hematological/biochemical response to any of the treatment given. Despite all aggressive measures, he had a continuous downhill course and succumbed to his illness on 21^st^ day of admission. We believe, had the patient been administered Eculizumab, the result would have been favourable.

38 cases have been reported in the past as per literature since 1978 [3–6]. Characteristics with respect to patient demography, etiology, laboratory & biochemical parameters, treatment offered and the outcome of the patient of all the cases reported so far has been compiled in Table 2. Atypical HUS has occurred in Acute Pancreatitis of varied etiology, inferring no specific association of aHUS with any particular etiology of AP. The short median interval from onset of Acute Pancreatitis to occurrence of HUS suggests causal association, in the absence of other known potential triggers for aHUS. Likely pathogenesis being profound inflammatory response associated with Severe AP leading to compliment hyperactivation mediated endothelial damage and subsequently its consequences.

**Table 2:** Characteristics of previously reported similar cases.

S. No	Year	Age, Sex	Etiology	Hb g/dl	Platelet 10^3^/uL	Creatinine mg/dl	Treatment	Renal Replacement Therapy	Hematological remission	Renal outcome	Death
1	1978	18, M	Idiopathic	10.6	2	2.3	SPLENECTOMY	No	Yes	CR	NO
2	1981	32, M	Alcohol	7.7	24	5.8	FLUIDS	Yes	Yes	CR	No
3	1989	55, F	Idiopathic	8.0	24	1.7	PLEX	No	Yes	CR	No
4	1991	36, M	Alcohol	9.2	45	-	-	-	-	-	-
5	1992	55, M	GSD	10	20	7.9	PLEX	Yes	Yes	CR	No
6	1992	48, M	Alcohol	8	40	2.1	CS	No	No	-	Yes
7	1995	18, M	Alcohol	6	30	15.0	PLEX	Yes	Yes	CR	No
8	1997	25, M	Alcohol	5	53	5.3	PLEX	-	-	-	-
9	1997	23, M	Alcohol	7.6	45	6.9	PLEX, CS	Yes	Yes	CR	NO
10	1998	28, F	Idiopathic	9.2	9	1.3	-	-	-	-	-
11	1998	65, M	GSD	10.3	32	2.2	PLEX	No	Yes	CR	No
12	1998	37, M	Alcohol	11.7	22	4.8	PLEX	No	Yes	CR	No
13	2000	70, M	Idiopathic	7.7	22	2.4	-	-	-	-	-
14	2000	30, M	Alcohol	7.6	63	8.3	PLEX, CS, IVIg	No	Yes	CR	No
15	2002	38, M	Alcohol	-	28	-	PLEX	Yes	Yes	CR	No
16	2002	35, M	ERCP	5.3	15	2.2	PLEX, CS	No	Yes	CR	No
17	2002	58, M	Alcohol	8.3	14	3.2	PLEX	Yes	Yes	CR	No
18	2003	38, F	Alcohol	9	24	7.7	PLEX	Yes	Yes	CR	No
19	2004	35, M	Alcohol	4.6	30	1.45	PLEX	No	No	CR	No
20	2004	33, M	Alcohol	5.8	90	7.0	PLEX	Yes	-	-	-
21	2005	55, F	ERCP	12.1	50	-	PLEX, RTX	Yes	Yes	-	No
22	2005	19, M	GSD	8.8	32	6.7	PLEX	Yes	Yes	CR	No
23	2005	43, M	Alcohol	10.1	53	2.0	-	No	Yes	CR	No
24	2005	37, F	Sarcoidosis	9.6	74	4.1	PLEX	No	Yes	CR	No
25	2006	35, F	GSD	8.0	35	2.1	PLEX	No	Yes	CR	No
26	2010	74, M	Idiopathic	6.4	20	6.4	PLEX	Yes	Yes	PR	No
27	2011	23, M	ERCP	8.7	24	1.7	PLEX	No	Yes	CR	No
28	2011	40, F	Idiopathic	5.9	11	5.7	PLEX	No	Yes	CR	No
29	2014	38, F	Alcohol	8.8	32	1.45	PLEX	Yes	Yes	CR	No
30	2016	21, M	ERCP	6.2	7.0	1.6	PLEX, ECZ	No	Yes	CR	No
31	2017	61, F	Alcohol	9.3	25	1.8	PLEX	No	Yes	PR	No
32	2019	37, M	Alcohol	7.3	28	7.1	ECZ	No	Yes	CR	No
33	2022	17, F	GSD	6.6	16	2.98	PLEX	Yes	Yes	CR	No
34	2023	68, M	Idiopathic	13.8	121	1.11	Ulinastatin	No	Yes	CR	No

Dash represent data not available; GSD, gall stone disease; PLEX, plasma exchange; CS, corticosteroids; ECZ, Eculizumab; IVIg, Intravenous Immunoglobulin; RTX, rituximab; M, male; F, female; CR, complete recovery; PR, partial recovery; Hb, hemoglobin. 4 cases reported in manuscript by Le Clech et al. [6] are not included.

We suggest long term Eculizumab with apt supportive care as the mainstay of treatment for aHUS in AP and Exchange Plasmapheresis with IVIg or Corticosteroids as second line alternative therapy. Rituximab can be used in refractory or relapsing HUS cases.

## Conclusion

Hemolytic Uremic Syndrome as a complication of Acute Pancreatitis is a rare presentation. Previously reported cases and this case highlights the possibility of aHUS secondary to Acute Pancreatitis. This condition should be diagnosed well in time as it is associated with high mortality. Lack of treatment protocol for aHUS in Pancreatitis makes management decisions difficult, leading to poor outcomes. HUS itself carries high mortality reaching as high up to 25%, but the overall outcome in the so far reported cases has been positive. This might be because of the under reporting of cases with unfavourable outcome. This emphasizes the need for further studies and research to streamline the management in such kind of cases.

## References

[1] MW SAIF . DIC secondary to acute pancreatitis. Clin Lab Haematol 2005;27:278–82. 10.1111/j.1365-2257.2005.00697.x 16048498

[2] Swisher KK, Doan JT, Vesely SK. et al. Pancreatitis preceding acute episodes of thrombotic thrombocytopenic purpura-hemolytic uremic syndrome: report of five patients with a systematic review of published reports. Haematologica 2007;92:936–43. 10.3324/haematol.10963 17606444

[3] Sandino-Pérez J, Gutiérrez E, Caravaca-Fontán F. et al. Haemolytic uraemic syndrome associated with pancreatitis: report of four cases and review of the literature. Clin Kidney J 2021;14:1713–3. 10.1093/ckj/sfab051 34252172 PMC8264306

[4] Taner S, Karaçay IET, Arslan İ. Acute pancreatitis complicated by hemolytic uremic syndrome: a pediatric case. Egyptian Pediatric association. Gazette 2022;70:43. 10.1186/s43054-022-00140-z

[5] Kajiyama T, Fukuda M, Rikitake Y. et al. Atypical Hemolytic uremic syndrome secondary to pancreatitis: a case report. Cureus 2023;15:e35434. 10.7759/cureus.35434 36994293 PMC10041130

[6] Le Clech A, Simon-Tillaux N, Provôt F. et al. Atypical and secondary hemolytic uremic syndromes have a distinct presentation and no common genetic risk factors. Kidney Int 2019;95:1443–52. 10.1016/j.kint.2019.01.023 30982675

[7] Noris M, Remuzzi G. Hemolytic uremic syndrome. J Am Soc Nephrol 2005;16:1035–50. 10.1681/ASN.2004100861 15728781

[8] Zimmerhackl L, Besbas N, Jungraithmayr T. et al. Epidemiology, clinical presentation, and pathophysiology of atypical and recurrent Hemolytic uremic syndrome. Semin Thromb Hemost 2006;32:113–20. 10.1055/s-2006-939767 16575686

[9] Karpman D, Loos S, Tati R. et al. Haemolytic uraemic syndrome. J Intern Med 2017;281:123–48. 10.1111/joim.12546 27723152

[10] Noris M, Mescia F, Remuzzi G. STEC-HUS, atypical HUS and TTP are all diseases of complement activation. Nat Rev Nephrol 2012;8:622–33. 10.1038/nrneph.2012.195 22986360

[11] Bernardo A, Ball C, Nolasco L. et al. Effects of inflammatory cytokines on the release and cleavage of the endothelial cell–derived ultralarge von Willebrand factor multimers under flow. Blood 2004;104:100–6. 10.1182/blood-2004-01-0107 15026315

[12] Caprioli J, Noris M, Brioschi S. et al. Genetics of HUS: the impact of MCP, CFH, and IF mutations on clinical presentation, response to treatment, and outcome. Blood 2006;108:1267–79. 10.1182/blood-2005-10-007252 16621965 PMC1895874

[13] Chang JC, Kathula SK. Various clinical manifestations in patients with thrombotic Microangiopathy. J Investig Med 2002;50:201–6. 10.2310/6650.2002.33434 12033285

[14] Gourley BL, Mesa H, Gupta P. Rapid and complete resolution of chemotherapy-induced thrombotic thrombocytopenic purpura/hemolytic uremic syndrome (TTP/HUS) with rituximab. Cancer Chemother Pharmacol 2010;65:1001–4. 10.1007/s00280-010-1258-4 20119714

